# A molecular analysis of the *GBA* gene in Caucasian South Africans with Parkinson's disease

**DOI:** 10.1002/mgg3.267

**Published:** 2017-02-08

**Authors:** Melinda Barkhuizen, David G. Anderson, Francois H. van der Westhuizen, Anne F. Grobler

**Affiliations:** ^1^ DST/NWU Preclinical Drug Development PlatformNorth‐West UniversityPotchefstroom2520South Africa; ^2^ Department of PaediatricsSchool for Mental Health and NeuroscienceMaastricht UniversityMaastricht6229The Netherlands; ^3^ Department of NeurologyUniversity of the Witwatersrand Donald Gordon Medical CentreJohannesburg2193South Africa; ^4^ Human MetabolomicsNorth‐West UniversityPotchefstroom2520South Africa

**Keywords:** Afrikaner, Gaucher disease, genetic screening, glucocerebrosidase, modeling, Parkinson's disease

## Abstract

**Background:**

The molecular basis of Parkinson's disease in South African population groups remains elusive. To date, substitutions in the *GBA* gene are the most common large‐effect genetic risk factor for Parkinson's disease. The primary objective of this study was to determine the prevalence of *GBA* substitutions in South Africans with idiopathic Parkinson's disease.

**Methods:**

Participants were recruited from tertiary hospitals in the Gauteng Province in South Africa. All participants were screened for substitutions in *GBA* exon 8‐11 and the full coding region was analysed in 20 participants. Peripheral *β*‐glucocerebrosidase enzymatic activity of *GBA*‐carriers was measured in mixed leukocytes.

**Results:**

Of 105 Caucasian Parkinson's disease participants (82.7% Afrikaner) with an average age of disease onset of 61.9 ± 12.2 years and 40 controls (age 73.4 ± 12.4 years) were included. Heterozygous *GBA* substitutions were identified in 12.38% of affected participants (p.G35A, p.E326K, p.I368T, p.T369M, p.N370S, p.P387L and p.K441N) and 5.00% of controls (p.E326K and p.T369M). The substitutions ranged from predicted benign to moderately damaging; with p.E326K and p.T369M most prevalent, followed by the Afrikaner Gaucher disease substitution p.P387L. Severe Gaucher disease mutations, like p.L444P, were absent in this cohort. Enzyme activity analysis revealed a nonsignificant reduction in the *GBA*‐Parkinson's disease individuals (14.49 ± 2.30 nmol/h/mg protein vs. 15.98 ± 3.06 nmol/h/mg in control samples). *GBA* substitutions occur in both young‐onset and late‐onset Parkinson's cases in the cohort.

**Conclusion:**

Mild *GBA* substitutions that may not cause Gaucher disease were a common risk factor for Parkinson's disease in the participant group.

## Introduction

Parkinson's disease (PD) is globally the 2nd most common neurodegenerative disorder. It is characterized by a loss of dopaminergic neurons in the substantia nigra. The formation of abnormal intraneuronal aggregates, termed Lewy bodies, is the main neuropathological hallmark of PD. Lewy bodies predominantly consist of insoluble *α*‐synuclein (Dauer and Przedborski 2003). The etiology of PD remains largely unknown. Classic Mendelian genes account for <10% of all cases (Lesage 2015). Multifactorial interactions between genetic risk factors and environmental exposures likely account for the majority of PD cases observed (Bekris et al. 2010). Cumulative genetic risk reduces the age‐of‐onset of PD. Meta‐analyses of genome‐wide association studies have identified 28 risk loci associated with PD (Nalls et al. 2015). Of these, the most statistically significant signals associated are common substitutions located close to the *SNCA* (OMIM: 163890)*, LRRK2* (OMIM: 609007)*, MAPT* (OMIM: 157140) genes and low‐frequency coding substitutions in the *GBA* (OMIM: 606463) gene (Benitez et al. 2016).

*GBA* substitutions are the most common large‐effect risk factor for PD globally. The *GBA* gene encodes the lysosomal hydrolase *β*‐glucocerebrosidase. Homozygous or compound heterozygous *GBA* substitutions cause the lysosomal storage disorder Gaucher disease (GD), whilst both homozygous and heterozygous substitutions increase PD susceptibility (Sidransky et al. 2009). *GBA* substitutions have been reported in 10.7–31.3% of Ashkenazi Jewish PD cases and in 2.3 – 9.4% of PD cases from other ethnic origins (Sidransky and Lopez 2012).

The *GBA* gene is located on chromosome 1q22. The gene consists of 11 exons and 10 introns spanning 7.6‐kb of sequence. A highly homologous pseudogene is located 16 kb downstream. Almost 300 unique *GBA* substitutions have been identified in patients with GD, including missense, nonsense, and frame‐shift substitutions as well as insertions, deletions, and complex alleles (Hruska et al. 2008). Approximately 70% of all the reported substitutions, including the two most common pathogenic substitutions, p.N370S and p.L444P, are located in exons 9‐10 of the *GBA* gene (Asselta et al. 2014).

The *β*‐glucocerebrosidase protein has a direct interaction with *α*‐synuclein (Yap et al. 2011). Lower *β*‐glucocerebrosidase activity has been reported in the brain regions affected by *α*‐synuclein pathology – even in sporadic PD cases without *GBA*‐substitutions (Gegg et al. 2012; Chiasserini et al. 2015). There is a feed‐forward loop between *β*‐glucocerebrosidase and *α*‐synuclein, where a reduction in enzymatic activity increases *α*‐synuclein accumulation and fibrillization. This further reduces the activity of the *β*‐glucocerebrosidase enzyme (Mazzulli et al. 2011). It is estimated that carriers of heterozygous *GBA* substitutions have a 1.5% probability of developing PD by 60 years of age and a 7.7% chance of developing PD by age 80 (Alcalay et al. 2014). Clinically, *GBA*‐associated PD cases resemble idiopathic PD. However, *GBA*‐PD patients are more prone to cognitive decline and they may have a younger average age‐of‐onset of PD (Alcalay et al. 2014; Brockmann et al. 2015). A loss of smell is also frequently reported by *GBA*‐substitution carriers, even in individuals without PD (Beavan et al. 2015).

The 2014 mid‐year population estimation reports indicate that South Africa has 55 million residents, of which 8.3% are Caucasian. Additionally, 8% of the total South African population is estimated to be older than 60 years (StatsSA, 2015). The Caucasian South African population predominantly consists of Afrikaans‐speaking individuals (63%) and English‐speaking individuals (StatsSA, 2012). The Afrikaans‐speaking Caucasian population – hereafter called the Afrikaners – is a unique ethnic group in South Africa with well‐documented ancestral records spanning a period of over 350 years. They are mainly descended from Dutch, German and French settlers that colonized RSA in the 17th and 18th centuries. There are also minor genetic contributions by British descendants and non‐European slaves (Greeff 2007). They have a relatively small gene pool, and several founder effects exist within the population. Previous genealogical research has shown that 40/48 Afrikaner families with PD can be traced back to a single founder couple (Geldenhuys et al. 2014). Prior genetic studies in South Africa have concluded that the known Mendelian PD genes are rare in South African population groups (Bardien et al. 2009, 2010; Keyser et al. 2009, 2010a,b, 2011; Haylett et al. 2012; Blanckenberg et al. 2014). However, the frequencies of genetic susceptibility substitutions in the local population groups have not yet been established. In this study, we explored the frequency of *GBA*‐substitutions in a Caucasian South African cohort diagnosed with PD. The possible pathogenicity of the substitutions was assessed with computational prediction algorithms, *β*‐glucocerebrosidase enzymatic activity analysis and through modeling the positions of these substitutions relative to the interaction interface between the *β*‐glucocerebrosidase and *α*‐synuclein proteins.

## Materials and Methods

### Ethical compliance

This project was conducted under ethics approval nr. NWU‐00051‐14‐A1 of the Health Research Ethics Committee of the North‐West University in South Africa.

### Participant selection

Hundred‐and‐five Caucasian South African PD patients were recruited through referring neurologists at tertiary hospitals in the Gauteng province of South Africa. All participants fulfilled the UK PD Society Brain Bank research criteria for diagnosis of PD (Gibb and Lees 1988) as assessed by a registered neurologist. The participants were all at least second‐generation South Africans.

Forty non‐related aged Caucasian control participants were recruited through medical practitioners from the Gauteng province in South Africa. Controls were excluded if they had a positive family history of PD or GD, or if they reported having more than one of the cardinal signs of PD. All participants provided written informed consent.

### Sequencing and data analysis

PCR amplification of the exon 8‐11 fragment of *GBA*, followed by Sanger sequencing of the exons and exon‐flanking regions was done in all PD‐participants and controls with previously described primers (Sato et al. 2005). In addition, in twenty PD‐participants, without mutations in exon 8‐11, the remaining *GBA* exons were screened in fragments from exon 1‐5 and exon 5‐7 with primers previously described by Stone et al. (2000). Direct sequencing was performed using the BigDye Terminator Sequence Ready Reaction kit version 3.1 (Applied Biosystems, South San Francisco, CA, USA) and analyzed on a 3130 × l Genetic Analyzer (Applied Biosystems, South San Francisco, CA, USA) at Inqaba Biotechnical Industries (Pretoria, South Africa). CLC Main Workbench version 7.6.2 software was used for the analysis of the sequencing electropherograms. The amino acid positions are described using the classic *GBA* nomenclature based on the amino acid position in the mature enzyme. This system omits the first 39 amino acid residues of reference sequence NP_000148.2 (Hruska et al. 2008). Samples with nonsynonymous substitutions were resequenced for confirmation.

The pathogenicity of nonsynonymous substitutions was predicted with seven different online algorithms – six function prediction scores (MutPred, MutationTaster‐2, Polyphen‐2, PhD‐SNP, SIFT, SNP&GO) and an ensemble score (CADD). Polyphen‐2 evaluates eight protein sequence features, three protein structure features (scores >0.5 are deleterious), SIFT assesses protein sequence conservation among homologs (scores >0.95 are deleterious), MutPred combines the SIFT score with an evaluation of 14 structural and functional properties (scores >0.5 are deleterious); MutationTaster‐2 evaluates DNA sequence conservation, splice site prediction, mRNA stability prediction and protein feature annotations (scores >0.5 are deleterious); PhD‐SNP and SNP&GO use a support vector machine classification based on the protein sequence and profile and the CADD‐algorithm uses distinct substitution annotation retrieved from Ensembl Variant Effect Predictor, data from the ENCODE project and information from UCSC genome browser tracks (scores >15 are deleterious) (Frousios et al. 2013; Dong et al. 2015).

### Enzyme activity analysis

The *β*‐glucocerebrosidase activity of extracted mixed leukocytes was measured with a previously described assay that uses 4‐methylumbelliferyl *β*‐glucopyranoside (4‐MUG) as a substrate in the presence of sodium taurocholate (1.88 g/L) (Nakagawa et al. 1982). The enzymatic assays were performed by Mr Iain Sinclair at the National Health Laboratory Service (Braamfontein, South Africa) as part of a Gaucher disease testing protocol.

### Molecular modeling

p*K*_*a*_ values for *β*‐glucocerebrosidase were set at 5.5 and 7.4 with PROPKA 2.0. A peptide consisting of residues 115–140 of *α*‐synuclein was docked interactively and minimized using Maestro/PIPER (Schrödinger Inc.) (Kozakov et al. 2006) to the *β*‐glucocerebrosidase x‐ray structure (PDB ID: 3GXM, chain C), similar to predictions previously conducted by Yap et al. (2011). Interactions were analyzed with the protein–protein interaction analysis tools and the interaction fingerprint analysis of Maestro (Schrödinger Inc.). Figures were made with PyMOL (Schrödinger Inc.).

### Statistical analysis

For differences in the prevalence of *GBA* mutations between PD and control participants, a chi‐square test was used. Enzymatic activity values were assessed for normality with a Shapiro–Wilk test and statistical significance was calculated with a *t*‐test. Statistical significance was set at *P*‐values smaller than 0.05.

## Results

### Participant characteristics

The affected PD participants had an average age‐of‐onset 61.9 ± 12.2 years with average disease duration of 7.5 ± 6.8 years prior to participating in this study. The gender ratio was 1:1.8 (female: male) and 82.7% of the participants were of self‐reported Afrikaner descent. The remainder was English‐speaking Caucasian South Africans. None of the participants reported Ashkenazi Jewish Ancestry.

The control participants had an average age of 73.4 ± 12.4 years. We preferentially selected a neurologically normal older control population to reduce the amount of participants that may later develop PD. The controls were predominantly of self‐reported Afrikaner descent (65%) and the gender ratio of the controls was 1:1.8 (male: female).

### *GBA* substitution prevalence

Nonsynonymous heterozygous *GBA* substitutions (including p.E326K and p.T369M) were identified in 12.4% of the PD participants and 5.0% of controls (*P* > 0.05). Putative GD‐causing substitutions were identified in 5.7% of PD participants and 0% of controls (*P* > 0.05), similar to frequencies previously reported in non‐Ashkenazi Jewish PD patients (Sidransky et al. 2009). The remainder of substitutions consisted of the non‐GD causing polymorphisms: p.E326K and p.T369M. In the exon 8‐11 fragment we identified: p.E326K (4.8% of PD‐participants, 2.5% of controls), p.I368T (1.0% of PD‐participants), p.T369M (1.9% of PD‐participants, 2.5% of controls), p.N370S (1.0% of PD‐participants), p.P387L (1.9% of PD‐participants) and p.K441N (1.0% of PD‐participants). Analysis of the remainder of the exon‐flanking regions in a subset of participants identified one additional substitution, p.G35A (5.0% of the subset of PD‐participants). The characteristics of the *GBA*‐substitution carriers are listed in Table 1. Synonymous and intronic substitutions identified are listed in Table S1.

**Table 1:** Nonsynonymous substitutions identified

Participant nr.	Exon	Substitution (traditional nomenclature)^a^	Substitution (according to reference sequence)^b^	rs number	Age‐of‐onset	Current age	Gender	Ethnicity	Family history of PD
**Putative GD‐causing mutations**									
PD participants									
1	3	p.G35A	p.G74A	rs371592589	71	76	M	Afrikaner	2nd degree
2	8	p.I368T	p.I407T	–	65	67	M	English	None
3	9	p.N370S	p.N409S	rs76763715	18	51	M	Afrikaner	None
4	9	p.P387L	p.P426L	–	38	53	M	Afrikaner	None
5	9	p.P387L	p.P426L	–	77	80	M	Afrikaner	None
6	10	p.K441N	p.K480N	–	42	57	F	Afrikaner	None
**Polymorphisms**									
PD participants									
7	8	p.E326K	p.E365K	rs2230288	24	37	M	Afrikaner	2nd degree
8	8	p.E326K	p.E365K	rs2230288	58	73	M	Afrikaner	None
9	8	p.E326K	p.E365K	rs2230288	60	72	F	Afrikaner	None
10	8	p.E326K	p.E365K	rs2230288	63	64	M	Afrikaner	None
11	8	p.E326K	p.E365K	rs2230288	80	84	F	Afrikaner	1st degree
12	8	p.T369M	p.T408M	rs75548401	65	71	M	Afrikaner	None
13	8	p.T369M	p.T408M	rs75548401	75	81	F	Afrikaner	None
Controls									
1	8	p.E326K	p.E365K	rs2230288	–	95	F	Afrikaner	None
2	8	p.T369M	p.T408M	rs75548401	–	81	F	Afrikaner	None

a Traditional protein numbering, which omits the first 39 amino acid residues.

b Protein numbering according to reference sequence NP_000148.2.

### Molecular modeling

To shed more light on the possible pathological impact of the substitutions identified in the study, the influence that these amino acid substitutions could have on the binding interface between *β*‐glucocerebrosidase and *α*‐synuclein was investigated with molecular modeling.

**Figure 1:**
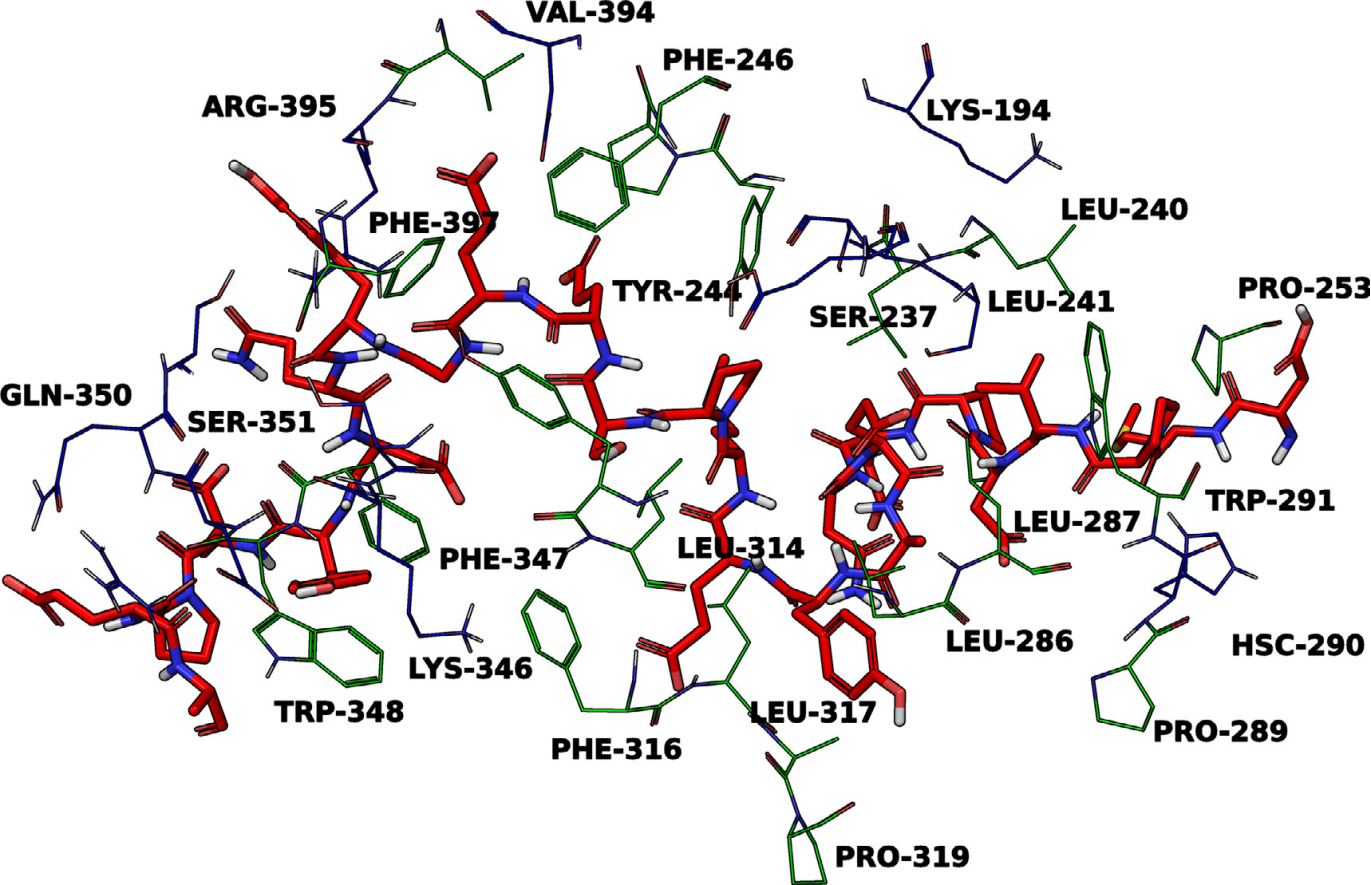
Important interactions between glucocerebrosidase and *α*‐synuclein at pH 5.5.Image guide: *α*‐synuclein residues 114‐140 (red) and selected polar and charged residues (blue), hydrophobic and aromatic residues (green) of *β*‐glucocerebrosidase 3GXM chain C.

The interactions between the wild‐type *β*‐glucocerebrosidase and wild‐type *α*‐synuclein proteins were determined first to determine whether any altered amino acids are directly involved in the protein–protein interactions (Fig. 1).

Next, wild‐type *α*‐synuclein was docked into a series of *β*‐glucocerebrosidase proteins with a single amino acid substitution. The substituted amino acids represent each variation identified in the participants. The modeling was performed to determine whether these substitutions alter the binding formation of the two proteins. Docking simulations were executed at pH 5.5 (lysosomal pH) and pH 7 (endoplasmic reticulum pH). These pHs were chosen since *β*‐glucocerebrosidase is secreted in the endoplasmic reticulum, but it has to be transported to the lysosomal compartment to exert its enzymatic activity. The p.N370S GD mutation causes early protein misfolding in the endoplasmic reticulum. This prevents the trafficking of *β*‐glucocerebrosidase to the lysosome, but does not reduce the intrinsic catalytic activity of the enzyme (Maegawa et al. 2009).The effect of the other substitutions identified in this study is less clear. Substitution of amino acids in *β*‐glucocerebrosidase only caused minor alterations were in the predicted‐binding conformation of *α*‐synuclein and none of the glucocerebrosidase substitutions were located directly on the interaction interface (Fig. S1).

The analysis of the molecular interaction fingerprints between the top 30 docked *α*‐synuclein poses in the substitution *β*‐glucocerebrosidase proteins, at both pH 5.5 and pH 7, showed that the residues that were predicted to contribute the majority of the binding energy between the two proteins remained fairly constant among all the glucocerebrosidase substitutions modeled. Thus, the amino acid substitution did not disrupt an important interaction between the two proteins.

In the majority of the molecular finger print analyses, *β*‐glucocerebrosidase residues Asp‐127, Lys‐194, Ser‐237, Asn‐238, Ser‐242, Glu‐340, Ser‐345, Gln‐350, Ser‐351 form polar interactions with the majority of *α*‐synuclein poses, regardless of pH. Residues Lys‐346, Glu‐349, Arg‐353, Asp‐358 and Arg‐395 form both polar and charged interactions with *α*‐synuclein at neutral and lysosomal pH. At pH 5.5 additional polar and charged interactions are contributed by His‐290 and Glu‐235 respectively. Residues Leu‐240, Leu‐241, Pro‐245, Pro‐253, Leu‐286, Leu‐287, Pro‐289, Leu‐314, Leu‐317, Ala‐318, Pro‐319, Val‐394 and Val‐398 form hydrophobic interactions, whilst residues Tyr‐244, Phe‐246, Trp‐291, Tyr‐313, Phe‐316, Phe‐347, Trp‐348 and Phe‐397 form both hydrophobic and aromatic interactions with *α*‐synuclein.

Lastly, the protein–protein interactions between the best wild‐type *α*‐synuclein (*α*S) pose and wild‐type *β*‐glucocerebrosidase (GC) were measured to identify individual residues of *α*‐synuclein that interacts with *β*‐glucocerebrosidase. At pH 5.5, *α*S Val‐118 interacts with GC residues 240‐241. *α*S Glu‐123 interacts with GC Ser‐237 and Leu‐241, *α*Syn residues 124‐125 interact with GC Leu‐286, *α*S Tyr‐125 also interacts with GC Leu‐317 and Ala‐318. *α*S residues 128 – 132 interacts with GCase residues 241, 244‐246, 313‐314. *α*S Glu‐131 also interacts with GC residues 127‐128 and 392. *α*S residues 131‐134 interact with GC 395‐397. *α*S residues 131‐140 interacts with GC 345‐353. The substitution amino acids in this study (Gly‐35, Glu‐326, Ile‐368, Thr‐369, Asn‐370, Pro‐387 and Lys‐441) do not appear to have an important role in the GCase‐*α*‐synuclein interaction.

### Impact of substitutions on *GBA* structure and function

**Figure 2:**
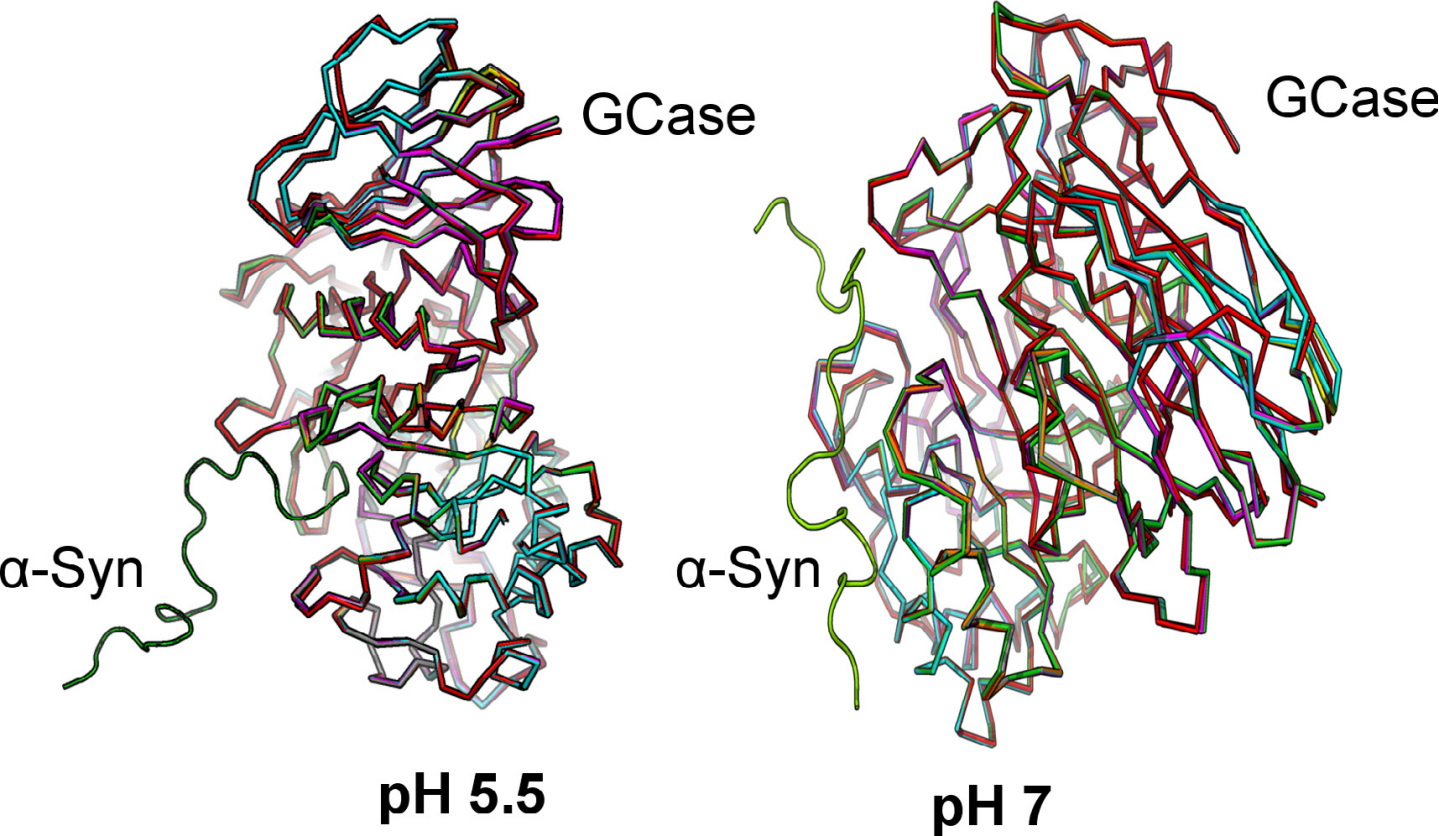
Overall conformation of the substituted *β*‐glucocerebrosidase receptor after minimization for docking with *α*‐synuclein at pH 5.5 and pH 7. Image guide: wild type = red, p.G35A = yellow, p.E326K = orange, p.I368T= grey, p.T369M = purple, p.N370S = lime, p.P387L = cyan, K441N = magenta. *α*‐synuclein is displayed as a green ribbon.

The putative structural impact of the amino‐acid substitution was analyzed *in silico* by visualization of the 3D conformation of the *β*‐glucocerebrosidase protein structure after energy minimization with Maestro/PIPER (Schrödinger Inc.) with *α*‐synuclein docked. The results of the minimization at pH 5.5 and pH 7 are shown in Fig. 2. The substitutions were not predicted to cause large changes in the overall *β*‐glucocerebrosidase protein folding, but it resulted in subtle differences in side‐chain orientation (Fig. S2). The molecular modeling did not identify a clear mechanism of pathogenicity of the amino‐acid substitutions.

Thus, we explored the predicted structural and functional consequences further with seven web‐based computational pathogenicity prediction tools (Table 2). The p.P387L and p.K441N substitutions were predicted to be benign by 7/7 tools, p.E326K and p.T369M were predicted to be benign by 4/7 tools. P.G35A and p.I368T were predicted to be damaging or possibly damaging by 5/7 tools and the common GD‐causing mutation, p.N370S, was predicted to be damaging by 7/7 algorithms. These results indicate that there were a mixture of probable‐benign substitutions and damaging substitutions identified in the participant group. The MutPred tool indicated the top five molecular features which are predicted to be influenced by amino‐acid substitution (Table S2) (Li et al. 2009). Among the actionable hypotheses, it was predicted that the p.E326K and p.I368T mutations could lead to a gain of a catalytic residue, p.T369M could lead to a loss of a catalytic residue and p.N370S and p.E326K could increase the glycosylation of the protein.

**Table 2:** Leukocyte *β*‐glucocerebrosidase activity and pathogenicity predictions of the identified substitutions

Predicted protein^a^^(^ b ^)^	GBA‐carrier enzymatic activity in nmol/h/mg protein (PD‐participant nr)	Intra‐assay control enzymatic activity nmol/h/mg protein	Scaled CADD v1.3 score (>15 = deleterious)	MutPred score (>0.5 = deleterious)	MutationTaster‐2 prediction	PhD‐SNP score	PolyPhen‐2 prediction (score)	SIFT Prediction (score)	SNP&GO score
p.E326K (p.E365K)	17.24 (10)	16.60	**17.33**	**0.521**	**Disease causing**	Neutral	Benign (0.15)	Tolerated (0.43)	Neutral
p.T369M (p.T408M)	17.55 (12)	18.89	**22.20**	**0.502**	Polymorphism	Neutral	Benign (0.275)	Tolerated (0.12)	**Disease**
	13.63 (13)	17.51							
p.G35A (p.G74A)	11.16 (1)	10.16	**23.50**	**0.809**	**Disease causing**	Neutral	**Possibly damaging (0.91)**	Tolerated (0.06)	**Disease**
p.I368T (p.I407T)	–	–	**26.60**	**0.847**	**Disease causing**	Neutral	**Probably damaging (0.977)**	Tolerated (0.07)	**Disease**
p.N370S (p.N409S)	12.29 (3)	11.65	**22.70**	**0.876**	**Disease causing**	**Disease**	**Possibly damaging (0.607)**	**Damaging (0.02)**	**Disease**
p.P387L (p.P426L)	16.96 (4)	17.51	8.40	0.440	Polymorphism	Neutral	Benign (0)	Tolerated (0.31)	Neutral
	14.17 (5)	18.89							
p.K441N (p.K480N)	12.95 (6)	16.60	10.04	0.441	Polymorphism	Neutral	Benign (0)	Tolerated (0.33)	Neutral

Damaging or possibly damaging scores are indicated in bold.

a Traditional protein numbering, which omits the first 39 amino acid residues.

b Protein numbering according to reference sequence NP_000148.2.

The in vitro effect of the substitutions on the catalytic activity of *β*‐glucocerebrosidase was measured in mixed leucocytes from a peripheral blood sample (Table 2). This measurement could not distinguish between enzymatic activity in participants and intra‐assay control samples in our study.

## Discussion

Our reported mutation frequencies are similar to reported frequencies persons with PD from non‐Ashkenazi Jewish descent (Sidransky et al. 2009). The *GBA* substitution spectrum in the South African population mainly consisted of mild damaging and possibly benign substitutions. We have only identified a single p.N370S carrier (rs76763715) and no p.L444P carriers. Globally, these 2 substitutions are considered to be the two most common *GBA* substitutions in both GD and PD. As a result, numerous studies have only screened for those 2 substitutions (Sidransky et al. 2009).

In our cohort, the most common nonsynonymous substitutions were the polymorphisms p.E326K (rs2230288) and p.T369M (rs75548401). These substitutions are known to cause a mild reduction in enzymatic activity and not cause GD, even in the homozygous state. However, p.E326K has been reported to predispose to PD and it is also a common PD‐associated substitution in other populations (Nichols et al. 2009; Horowitz et al. 2011; Duran et al. 2013). Similarly, the p.T369M substitution has previously been identified in PD cases (Clark et al. 2007; Nichols et al. 2009; Benitez et al. 2016). Whilst it is known that severe GD‐associated *GBA* substitutions increase the risk of cognitive decline (Alcalay et al. 2012; Winder‐Rhodes et al. 2013), recent evidence indicated that the p.E326K substitution also increases the risk of working memory/executive function decline and visuospatial memory impairment in PD (Mata et al. 2015).

The p.P387L substitution has previously been identified in an Afrikaner GD individual (Morar and Lane 1996). It was subsequently included in the GD genetic screening panel for Afrikaner individuals, but our computational predictions indicate that it may be a benign polymorphism. The p.G35A substitution (rs371592589) has been identified previously in a South African mixed ancestry individual with unknown PD or GD status. Another nonsynonymous substitution of the same amino acid, p.G35S, has been linked to GD (Rozenberg et al. 2006). The p.G35A carrier also had a 2nd degree relative with PD. This substitution was predicted to be damaging by the majority of algorithms.

We also report two novel *GBA* substitutions, p.I368T and p.K441N. The p.I368T substitution occurred in a late‐onset sporadic PD case of English South African ancestry. It was predicted to be damaging by the majority of algorithms. The p.K441N substitution occurred in a sporadic young‐onset Afrikaner participant. The substitution was predicted to be benign. Interestingly, modeling of the *α*‐synuclein – glucocerebrosidase interaction by Yap et al. (2011) has predicted that there is an electrostatic interaction between *β*‐glucocerebrosidase Lys‐441 and the negatively charged residues of *α*‐synuclein. Thus the p.K441N substitution could potentially cause PD by disrupting an interaction with *α*‐synuclein, rather than by reducing intrinsic enzymatic activity. We aimed to replicate their interaction between *β*‐glucocerebrosidase and *α*‐synuclein, but Lys‐441 did not contribute significantly to the protein–protein interaction in our simulations.

Since we only screened for exon 8‐11 in the majority of the cohort, it is possible that up to 30% of substitutions were not identified and that the true nonsynonymous substitution frequency could reach up to 16% of persons with PD. In the overall frequency data we only report the nonsynonymous substitution frequency. Previous computational analysis shows that the majority of the intronic and synonymous substitutions – as well as a few exonic substitutions, may alter splicing (Barkhuizen et al. 2016). However, this has not been verified in vitro and it would be worthwhile to pursue in the future with the rare intronic substitutions.

The peripheral *β*‐glucocerebrosidase enzymatic activity of PD patients has been reported to be lower than in controls and *GBA*‐heterozygotes had a lower enzymatic activity than noncarriers. These measurements were made with mass‐spectrometry of dried blood spots (Alcalay et al. 2015; Glickman et al. 2015). In our study, the difference in enzyme activity was only slightly lower than the control values and it did not reach statistical significance (*P* > 0.05). This could reflect that the substitution spectrum predominantly consists of mild substitutions or due to a small sample size. However, even the p.N370S heterozygote did not have a marked reduction in *β*‐glucocerebrosidase activity. It is likely that the 4‐methylumbelliferyl *β*‐glucopyranoside assay in leukocytes was not sensitive enough to identify individual *GBA*‐carriers for routine clinical use. This correlates with observations by Kim et al. (2016) who found that the leukocyte 4‐methylumbelliferyl *β*‐glucopyranoside assay cannot distinguish sporadic PD patients from controls in a cohort that was not genotyped for *GBA* substitutions. In a genotyped cohort, Ortega et al. (2016) observed similar enzymatic activity in *GBA‐*PD participants (16.0 nmol/h/ mg protein) than in our study (14.49 ± 2.30 nmol/h/mg protein). However, they observed much higher values in idiopathic PD (28.5 nmol/h/mg protein) and asymptomatic *GBA* carriers (25.5 nmol/h/mg protein), than in the intra‐assay control subjects in our study (15.98 ± 3.06 nmol/h/mg) and the controls a previous South African GD study (13.71 ± 2.85 nmol/h/mg) (Arndt et al. 2009) with similar methodology. Whether leucocyte *β*‐glucocerebrosidase activity can reliably identify *GBA*‐PD participants remains open for further testing, but in this study it did not distinguish *GBA*‐PD from control individuals.

The age‐of‐onset of PD in the cohort was divided, with *GBA* substitutions observed in both young‐and late‐onset cases. It is possible that confounding genetic factors drive the earlier age‐of‐onset in the young‐onset cases, particularly as the majority of the substitutions are predicted to be benign and these participants have not been tested for other PD‐related genes. Alternately, it is possible that the late‐onset participants carry a protective modifier substitution.

The contribution of *GBA* to PD in other South African population groups remains to be studied in the future. Genotyping of Black South Africans with Gaucher disease revealed substantial differences in the substitution spectrum in comparison to the Caucasian population groups (Arndt et al. 2009). The study did not manage to enrol sufficient amounts of Black African, Mixed Ancestry or Indian ethnicity PD patients to make definite conclusions about other South African population groups.

## Conclusion

Mild *GBA* substitutions may be a substantial risk factor for PD in the South African Caucasian populations. The prevalence of *GBA* substitutions in the South African Afrikaner community is higher than previously thought. Since *GBA* substitutions also increase the risk of cognitive decline, it may be clinically relevant to screen Afrikaner PD patients for *GBA* substitutions.

## Conflict of Interest

The authors declare that they have no conflict of interest to disclose.

## Supporting information


**Figure S1.** The top‐poses of the *α*‐synuclein side‐chain docked into substituted *β*‐glucocerebrosidase, shown in relation to the position of substitutions identified.

**Figure S2.** A magnified view of the conformation of selected substituted *β*‐glucocerebrosidase receptors at pH 5.5 docked with *α*‐synuclein.

**Table S1.** Synonymous and intronic substitutions identified.

**Table S2.** MutPred predictions of functional consequences of the substitutions (Li et al. 2009).
