# Supplementary figures and images for: A molecular analysis of the GBA gene in Caucasian South Africans with Parkinson's disease
Source: Mol Genet Genomic Med. 2017 Feb 8;5(2):147–56. doi: 10.1002/mgg3.267 (PMC5370228; doi:10.1002/mgg3.267)

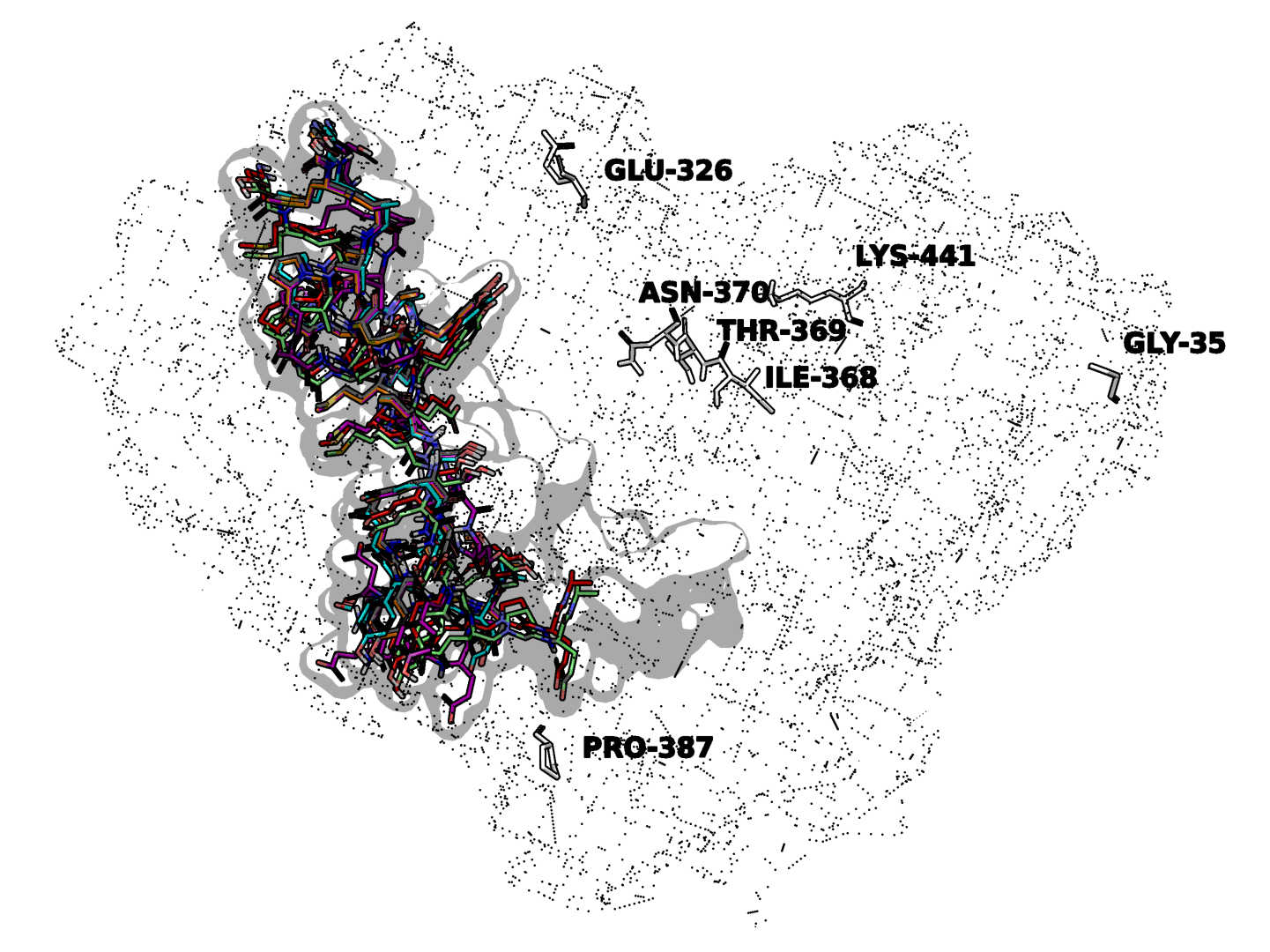

Supplement: Supplementary file 1 — Figure S1. The top‐poses of the α‐synuclein side‐chain docked into substituted β‐glucocerebrosidase, shown in relation to the position of substitutions identified. [file MGG3-5-147-s001.tif]

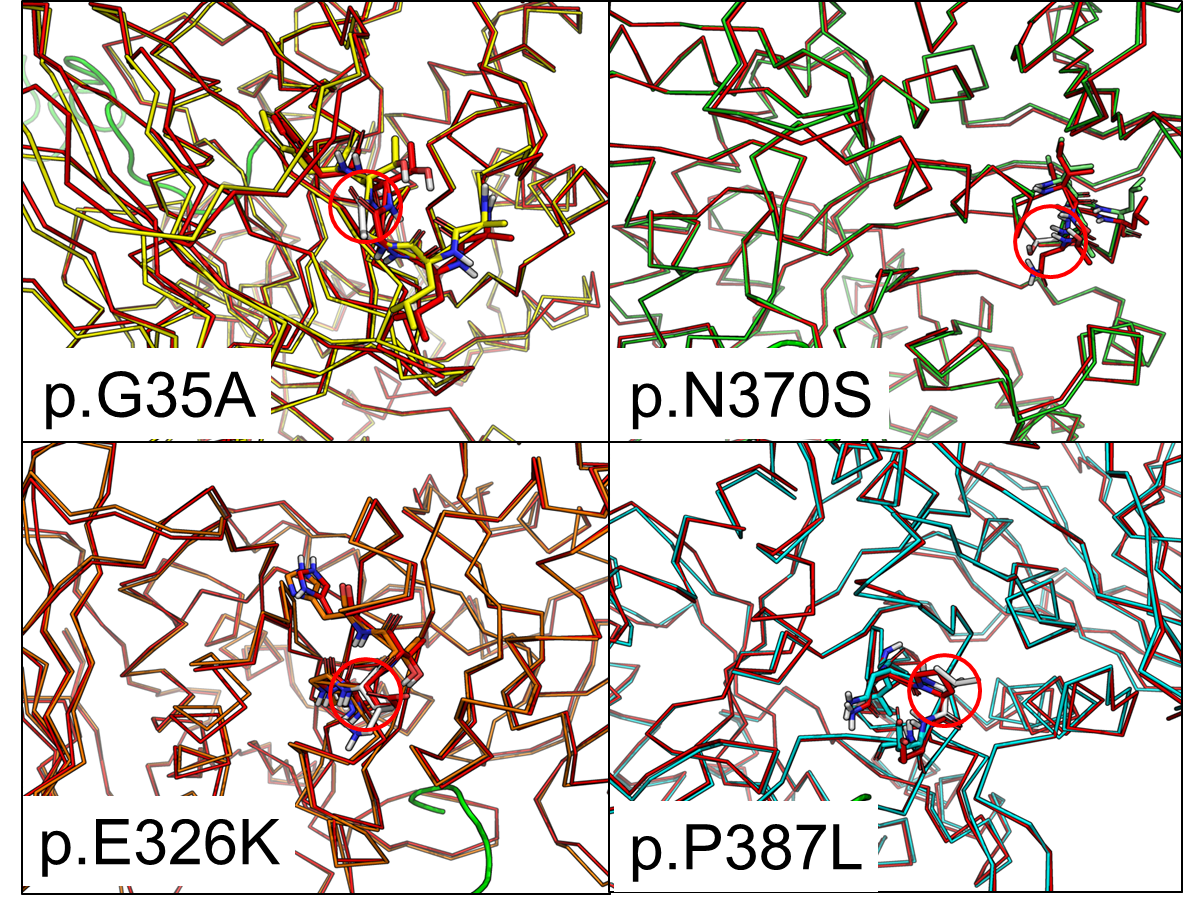

Supplement: Supplementary file 2 — Figure S2. A magnified view of the conformation of selected substituted β‐glucocerebrosidase receptors at pH 5.5 docked with α‐synuclein. [file MGG3-5-147-s002.tif]
