# Supplementary material for: A molecular analysis of the GBA gene in Caucasian South Africans with Parkinson's disease
Source: Mol Genet Genomic Med. 2017 Feb 8;5(2):147–56. doi: 10.1002/mgg3.267 (PMC5370228; doi:10.1002/mgg3.267)
Supplement: Supplementary file 3 — Table S1. Synonymous and intronic substitutions identified. [file MGG3-5-147-s003.docx]

Supplementary table 1 - Synonymous and intronic variants identified.

| Variant | rs number | % of patients | % of controls |
| --- | --- | --- | --- |
| c.1225-34C>A | rs3115534 | 93.3% | 100% |
| c.1388+141A>G | rs28373017 | 32.3% | 42.5% |
| c.1388+94G>A | rs12752133 | 6.7% | 2.5% |
| c.1506-10T>G | rs577529715 | 1.0% | 0.0% |
| c.1389-68T>C | rs143255568 | 2.9% | 0.0% |
| p.L249= | - | 1.0% | 0.0% |

cDNA numbering relative to reference sequence NM_000157.3 and starting at first nucleotide of translation initiation codon.
