# Supplementary material for: A molecular analysis of the GBA gene in Caucasian South Africans with Parkinson's disease
Source: Mol Genet Genomic Med. 2017 Feb 8;5(2):147–56. doi: 10.1002/mgg3.267 (PMC5370228; doi:10.1002/mgg3.267)
Supplement: Supplementary file 4 — Table S2. MutPred predictions of functional consequences of the substitutions (Li et al. 2009). [file MGG3-5-147-s004.docx]

Supplementary table 2 - MutPred predictions of functional consequences of the mutations (Li et al., 2009).

| **Mutation** | **Probability of deleterious mutation** | **MOLECULAR   MECHANISM**  **DISRUPTED** | | | **Top 5 features** |
| --- | --- | --- | --- | --- | --- |
|  |  | **Actionable Hypotheses** | **Confident Hypotheses** | **Very Confident Hypotheses** |  |
| p.E326K  (p.E365K) | 0.521 | Gain of glycosylation at E326 (P = 0.0429) Gain of catalytic residue at E326 (P = 0.0451) |  |  | Gain of glycosylation at E326 (P = 0.0429) Gain of catalytic residue at E326 (P = 0.0451) Gain of ubiquitination at E326 (P = 0.0539) Loss of helix (P = 0.1299) Loss of loop (P = 0.2237) |
| p.T369M  (p.T408M) | 0.502 | Loss of catalytic residue at T369 (P = 0.0205) |  |  | Loss of catalytic residue at T369 (P = 0.0205) Gain of helix (P = 0.2294) Gain of MoRF binding (P = 0.2299) Loss of glycosylation at S366 (P = 0.2477) Loss of disorder (P = 0.5389) |
| p.G35A (p.G74A) | 0.809 |  |  |  | Gain of catalytic residue at G35 (P = 0.0565) Loss of loop (P = 0.0804) Gain of helix (P = 0.0854) Loss of disorder (P = 0.1376) Gain of methylation at R39 (P = 0.1545) |
| p.I368T  (p.I407T) | 0.847 |  | Loss of stability (P = 0.0101)  Gain of catalytic residue at I368 (P = 0.0364) |  | Loss of stability (P = 0.0101)  Gain of catalytic residue at I368 (P = 0.0364)  Loss of helix (P = 0.0558)  Gain of disorder (P = 0.0562)  Gain of loop (P = 0.2045) |
| p.N370S  (p.N409S) | 0.876 |  | Gain of glycosylation at N370 (P = 0.0406) |  | Gain of glycosylation at N370 (P = 0.0406) Loss of stability (P = 0.1211) Gain of disorder (P = 0.2635) Loss of MoRF binding (P = 0.3213) Gain of catalytic residue at N370 (P = 0.3508) |
| p.P387L  (p.P426L) | 0.440 |  |  |  | Loss of disorder (P = 0.073) Loss of relative solvent accessibility (P = 0.114) Loss of helix (P = 0.1706) Loss of solvent accessibility (P = 0.1813) Gain of loop (P = 0.2754) |
| p.K441N  (p.K480N) | 0.441 |  |  |  | Loss of ubiquitination at K441 (P = 0.0074) Loss of sheet (P = 0.0817) Loss of disorder (P = 0.2055) Loss of methylation at K441 (P = 0.2525) Gain of catalytic residue at S439 (P = 0.259) |
